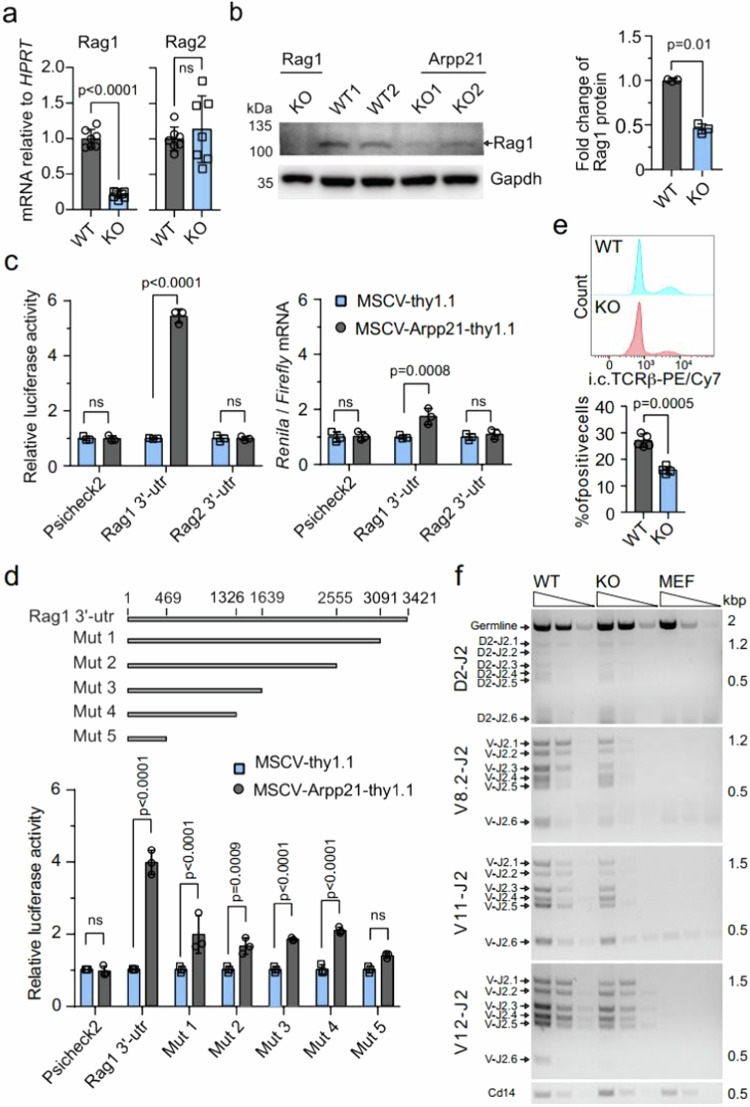# Correction to “The thymocyte-specific RNA-binding protein Arpp21 provides TCR repertoire diversity by binding to the 3’-UTR and promoting *Rag1* mRNA expression”

**DOI:** 10.1038/s41467-024-50610-8

**Published:** 2024-08-05

**Authors:** Meng Xu, Taku Ito-Kureha, Hyun-Seo Kang, Aleksandar Chernev, Timsse Raj, Kai P. Hoefig, Christine Hohn, Florian Giesert, Yinhu Wang, Wenliang Pan, Natalia Ziętara, Tobias Straub, Regina Feederle, Carolin Daniel, Barbara Adler, Julian König, Stefan Feske, George C. Tsokos, Wolfgang Wurst, Henning Urlaub, Michael Sattler, Jan Kisielow, F. Gregory Wulczyn, Marcin Łyszkiewicz, Vigo Heissmeyer

**Affiliations:** 1 Research Unit Molecular Immune Regulation, Molecular Targets and Therapeutics Center, Helmholtz Zentrum München, Munich, Germany; 2 Department of Integrated Traditional Chinese and Western Medicine, Union Hospital, Tongji Medical College, Huazhong University of Science and Technology, Wuhan, China; 3 Institute for Immunology, Biomedical Center (BMC), Faculty of Medicine, Ludwig-Maximilians-Universität in Munich, Planegg-Martinsried, Germany; 4 Institute of Structural Biology, Molecular Targets and Therapeutics Center, Helmholtz Zentrum München, Neuherberg, Germany; 5 Technical University of Munich, TUM School of Natural Sciences, Department of Bioscience and Bavarian NMR Center (BNMRZ), Garching, Germany; 6 Max Planck Institute for Multidisciplinary Sciences, Bioanalytical Mass Spectrometry, Göttingen, Germany; 7 Institute of Developmental Genetics, Helmholtz Zentrum München, Neuherberg, Germany; 8 Department of Pathology, New York University, Grossman School of Medicine, New York, NY USA; 9 Department of Medicine, Beth Israel Deaconess Medical Center, Harvard Medical School, Boston, MA USA; 10 Institute for Molecular Biology, Biomedical Center (BMC), Faculty of Medicine, Ludwig-Maximilians-Universität in Munich, Planegg-Martinsried, Germany; 11 Monoclonal Antibody Core Facility, German Research Center for Environmental Health, Neuherberg, Germany; 12 Research Unit Type 1 Diabetes Immunology, Helmholtz Diabetes Center at Helmholtz Zentrum München, Neuherberg, Germany; 13 German Center for Diabetes Research (DZD), Neuherberg, Germany; 14 Division of Clinical Pharmacology, Department of Medicine IV, Ludwig-Maximilians-Universität München, Munich, Germany; 15 Max von Pettenkofer Institute, Faculty of Medicine, Ludwig-Maximilians-Universität in Munich, Munich, Germany; 16 Institute of Molecular Biology (IMB), Mainz, Germany; 17 Chair of Developmental Genetics, Munich School of Life Sciences Weihenstephan, Technical University of Munich, Freising, Germany; 18 Munich Cluster of Systems Neurology (SyNergy), Munich, Germany; 19 German Center for Neurodegenerative Diseases (DZNE) site Munich, Munich, Germany; 20 University Medical Center Göttingen, Department of Clinical Chemistry, Bioanalytics Group, Göttingen, Germany; 21 Göttingen Center for Molecular Biosciences, Georg-August University Göttingen, Göttingen, Germany; 22 Cluster of Excellence ‘Multiscale Bioimaging: from Molecular Machines to Networks of Excitable Cells’ (MBExC), University of Göttingen, Göttingen, Germany; 23 Institute for Molecular Health Sciences, ETH Zürich, Zürich, Switzerland; 24 Institute for Integrative Neuroanatomie, Charite-Universitätsmedizin Berlin, Corporate Member of Freie Universität Berlin, Humboldt-Universität zu Berlin, and Berlin Institute of Health, Berlin, Germany; 25 Department of Pediatrics and Adolescent Medicine, University Medical Center Ulm, Ulm, Germany; 26 Present Address: Cancer Immunology and Immune Modulation, Boehringer Ingelheim Pharma GmbH & Co. KG, Biberach an der Riss, Germany; 27 Present Address: Repertoire Immune Medicines (Switzerland) AG, Schlieren, Switzerland

**Keywords:** VDJ recombination, Immunological disorders, T-cell receptor, Lymphocyte differentiation, Thymus

Correction to: *Nature Communications* 10.1038/s41467-024-46371-z, published online 11 March 2024

The original version of this Article contained errors in Fig. 6, in which the panel labels were misplaced, causing mismatches between the panels and legend. This has been corrected in both the PDF and HTML versions of the Article.