# Supplementary material for: The thymocyte-specific RNA-binding protein Arpp21 provides TCR repertoire diversity by binding to the 3’-UTR and promoting Rag1 mRNA expression
Source: Nat Commun. 2024 Mar 11;15:2194. doi: 10.1038/s41467-024-46371-z (PMC10928157; doi:10.1038/s41467-024-46371-z)
Supplement: Supplementary file 1 — Supplementary Information [file 41467_2024_46371_MOESM1_ESM.pdf]

**Inventory of Supplementary Materials**

1. **Supplementary Table 1**
2. **Supplementary Table 2**
3. **Supplementary Table 3**
4. **Supplementary Fig. 1**
5. **Supplementary Fig. 2**
6. **Supplementary Fig. 3**
7. **Supplementary Fig. 4**
8. **Supplementary Fig. 5**
9. **Supplementary Fig. 6**
10. **Supplementary Fig. 7**
11. **Supplementary Fig. 8**
12. **Supplementary Fig. 9**
13. **Supplementary Fig. 10**
14. **Supplementary Fig. 11**
15. **Supplementary Fig. 12**
16. **Uncropped Blots of Supplementary Fig.2a, b, e and Supplementary Fig. 3h and Supplementary Fig. 6d**

**Supplementary Table 1:** Primers for PCR amplification and cloning

| pRetroX-tight constructs | | |
|---|---|---|
| Gene name | Forward Primer | Reverse Primer |
| pRetroX-GFP | GCGGCCGCACTCGAGATATC | GGTCGACCCCTTGTACAGCT |
| Arpp21 | GCTGTACAAGGGGTCGACCTCTGAGCAAGGAGGAC | ATATCTCGAGTGCGGCCGCTCAGAACTTGACCTGC |
| Arpp21$^{short}$ | GAAAGTCACCAGGATCAGACTCTCTGAGCGGCCGCACTCGAG | CTCGAGTGCGGCCGCTCAGAGAGTCTGATCCTGGTGACTTTC |
| R3hdm1 | GCTGTACAAGGGGTCGACCAGGATGTCTGATATTG | ATATCTCGAGTGCGGCCGCTTACTGAGAACTTGCCC |
| R3hdm2 | GCTGTACAAGGGGTCGACCTCTAACAGTAACACTAC | ATATCTCGAGTGCGGCCGCCTATTGAGAGCTAGCTCG |
| R3hdm4 | GCTGTACAAGGGGTCGACCGTGGCTCTGGACAACT | ATATCTCGAGTGCGGCCGCTTGCTGGTCCAGGTAG |
| psiCHECK-2-3'UTR reporter constructs | | |
| psiCHECK-2 | GCGGCCGCTGGCCGCAATA | CTCGAGCGATCGCCTAGAATTACTG |
| Rag1 3'-UTR | ATTCTAGGCGATCGCTCGAGATAGGATCTCCACATAGAAGTTGGT | TTATTGCGGCCAGCGGCCGCTTTTCAGATCTTAAACTTTTATTCC |
| Rag2 3'-UTR | AGGCGATCGCTCGAGTTTAGCAAAAGCCCCTCAGACTCA | GCGGCCAGCGGCCGCGGAATATGAAAGTTTTCTT |
| Rag1 3'-UTR Mut1 | AGGCGATCGCTCGAGATAGGATCTCCACATAGAAGTT | GCGGCCAGCGGCCGCCTTACATAGATACTTAAAGAAAGAA |
| Rag1 3'-UTR Mut2 | AGGCGATCGCTCGAGATAGGATCTCCACATAGAAGTT | GCGGCCAGCGGCCGCCCAAGACACTTCTTAACCTCTCACC |
| Rag1 3'-UTR Mut3 | AGGCGATCGCTCGAGATAGGATCTCCACATAGAAGTT | GCGGCCAGCGGCCGCGACTGACAATTATGACCAATAAAA |
| Rag1 3'-UTR Mut4 | AGGCGATCGCTCGAGATAGGATCTCCACATAGAAGTT | GCGGCCAGCGGCCGCGCTGCTACTGTATAGACAACAGA |
| Rag1 3'-UTR Mut5 | AGGCGATCGCTCGAGATAGGATCTCCACATAGAAGTT | GCGGCCAGCGGCCGCTATGACCAAAAAATATGACATGGCA |

**Supplementary Table 2:** Primers and probes from the Universal Probe Library (UPL) system (Roche) used for RT-qPCR analyses.

| Name | Forward Primer | Reverse Primer | UPL probe |
|---|---|---|---|
| Arpp21 | CAGGAAAGCCTATTTTTGGACA | CTGAGCTGTCTCGGTGAGC | 1 |
| Rag1 | AGGCCTGTGGAGCAAGGTA | GCTCAGGGTAGACGGCAAG | 46 |
| Rag2 | TGCCAAAATAAGAAAGAGTATTTCAC | GGGACATTTTTGATTGTGAATAGG | 4 |
| Hprt | TCCTCCTCAGACCGCTTTT | CCTGGTTCATCATCGCTAATC | 95 |
| ActB | CTAAGGCCAACCGTGAAAAG | ACCAGAGGCATACAGGGACA | 64 |
| Renila | CGAGAACGCCGTGATTTT | GACGTGCCTCCACAGGTAG | 16 |
| Firefly | CAGAGCATGTACACATTCGTGA | CGAAGTCGTACTCGTTGAAGC | 16 |

**Supplementary Table 3:** Primers for TCRβ rearrangement

| Name | Sequence (5'-3') |
|---|---|
| D beta 2 | GTAGGCACCTGTGGGGAAGAAACT |
| V beta 8.2 | CTACCCCCTCTCAGACATCA |
| V beta 11 | TGCTGGTGTCATCCAAACACCTAG |
| V beta 12 | AGTTACCCAGACACCCAGACATGA |
| J beta 2 | TGAGAGCTGTCTCCTACTATCGATT |
| CD14 for | GCTCAAACTTTCAGAATCTAC |
| CD14 rev | AGTCAGTTCGTGGAGGCCGGAAATC |

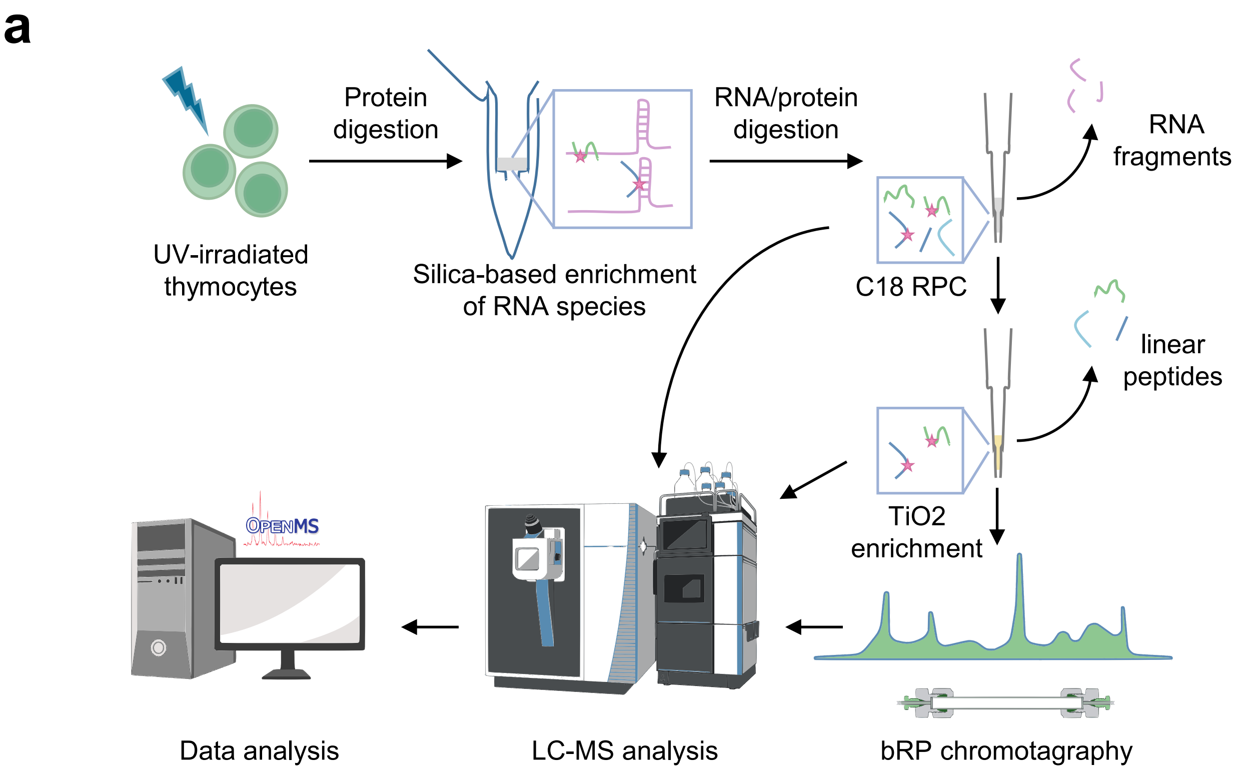


Supplementary Figure 1

**Supplementary Fig. 1. Workflow for direct identification of peptides with crosslinked RNA moieties.** Generation and enrichment of peptide-RNA heteroconjugates from cells. The irradiated cells were lysed and proteins digested with endoproteinase. RNA containing species, including crosslinked heteroconjugates were enriched over linear peptides by silica-based purification. The RNA moiety was digested and non-crosslinked nucleotides were depleted by C18 reversed-phase chromatography. An extra step of TiO2 purification and basic reversed-phase chromatography further diminished competing linear peptides and enabled thorough mass spectrometric analysis. The combination of enrichment and prefractionation steps resulted in identification of the crosslinked site in a significant number of proteins, commonly with single amino acid resolution. The Figure was created with BioRender.com.

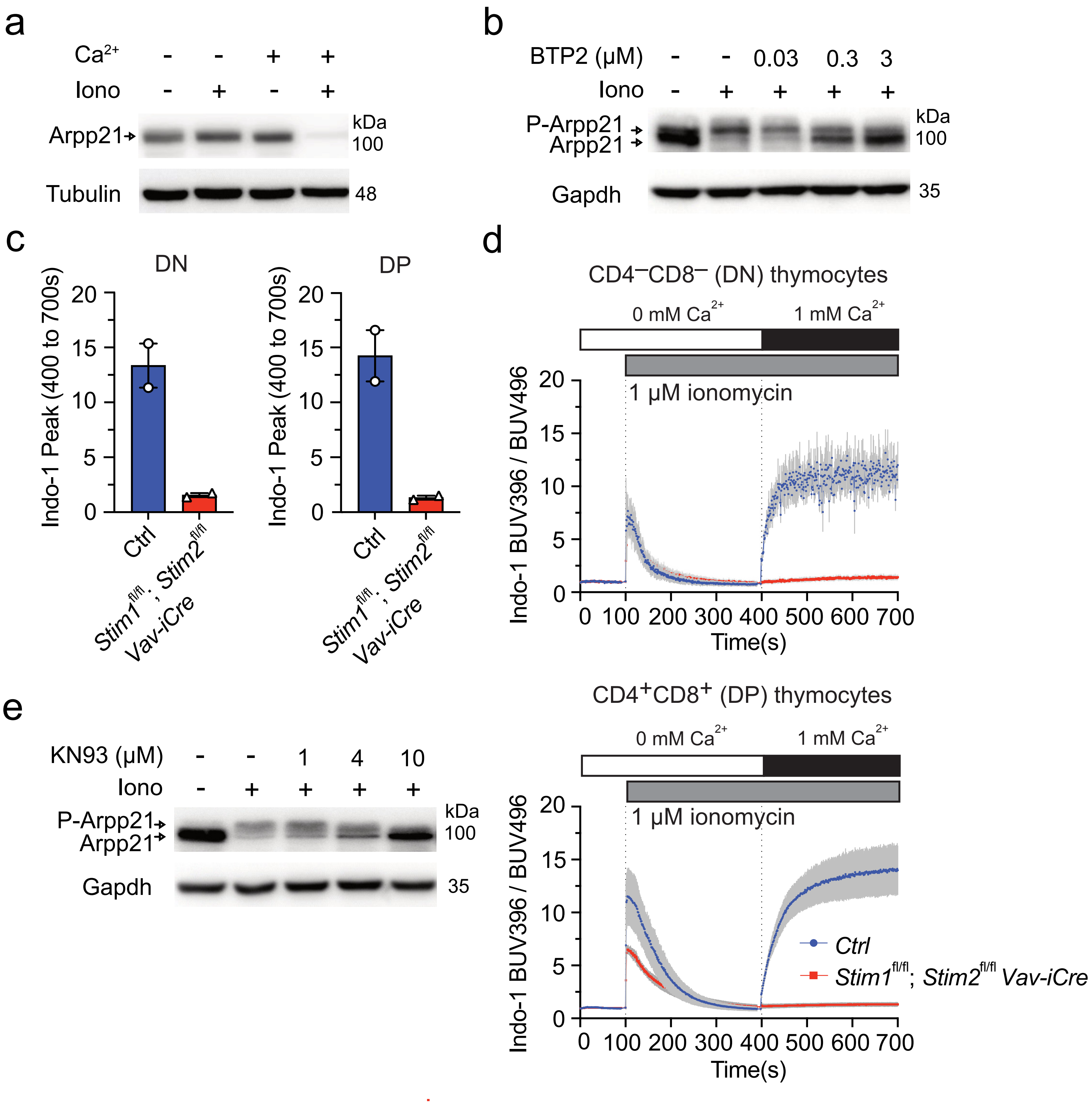


Supplementary Figure 2

**Supplementary Fig. 2. Inhibition of $Ca^{2+}$ signaling blocks phosphorylation and degradation of Arpp21 in thymocytes.** (a) WB analyses of Arpp21 expression in thymocytes incubated with Ringer buffer with or without $Ca^{2+}$. (b) Arpp21 and P-Arpp21 expression in thymocytes treated with BTP2 and ionomycin. (c-d) $Ca^{2+}$ measurements of SOCE in thymocytes from *Stim1*$^{fl/fl}$;*Stim2*$^{fl/fl}$;*Vav-iCre* (Stim1/Stim2 DKO) mice stimulated with ionomycin in $Ca^{2+}$-free buffer followed by readdition of 1 mM $Ca^{2+}$. (e) Immunoblot analyses of Arpp21 in thymocytes treated with KN-93 and ionomycin.

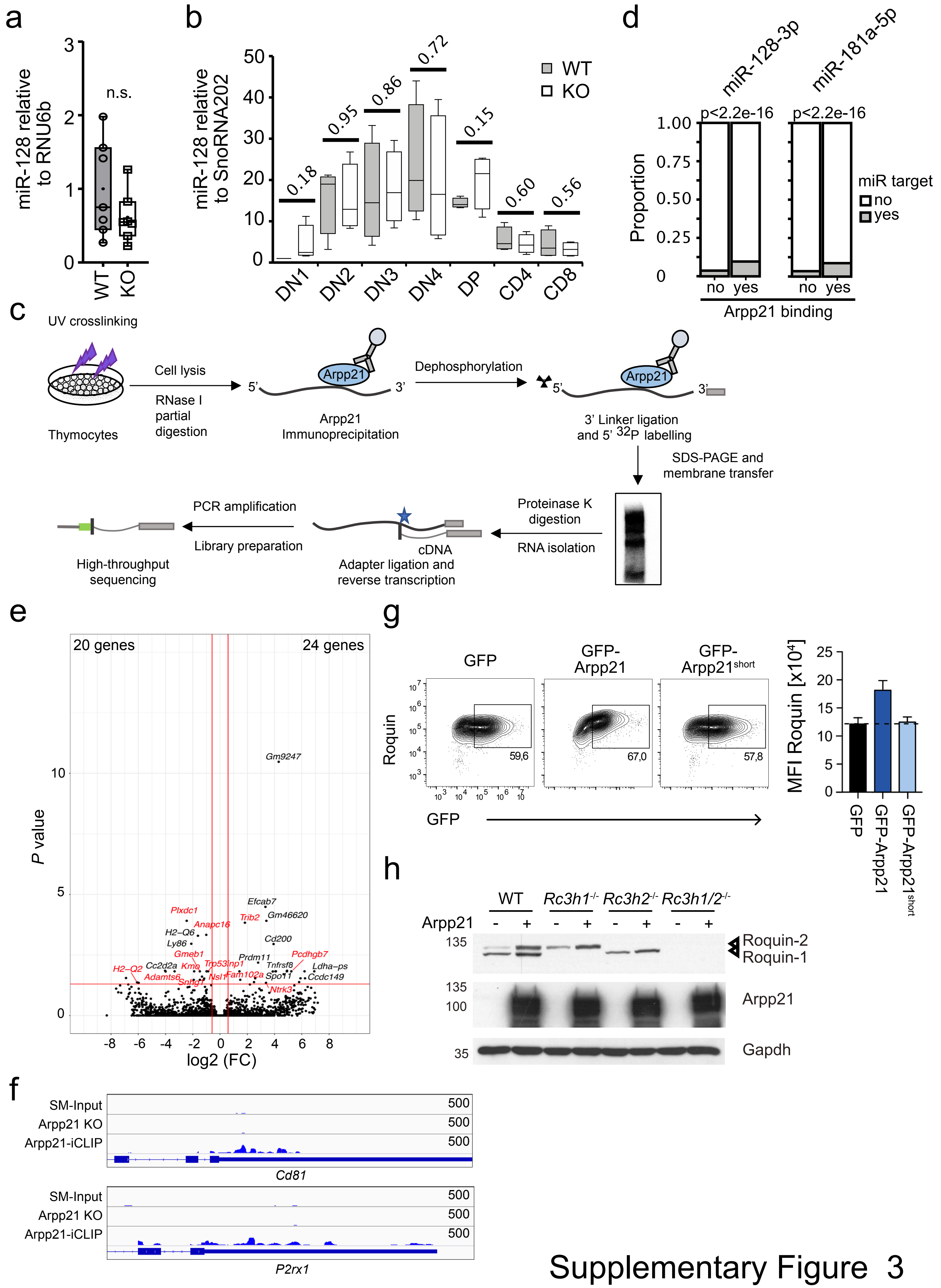


Supplementary Figure 3

**Supplementary Fig. 3. iCLIP identifies targets and binding sites of Arpp21 in the transcriptome of thymocytes.** (**a**) RT-qPCR analysis to determine the mature form of miR-128 as compared to the housekeeping gene RNU6b in thymocytes of WT and *Arpp21*$^{-/-}$ mice. Data are presented as mean values ± SD. n = 7 biological replicates, n.s. indicates not significant ($p$>0.05) as determined by Student t-test. (**b**) Quantification of mature miR-128 relative to SnoRNA202 in flow cytometry-sorted thymocytes from wildtype or Arpp21-deficient mice. n = 4 biological replicates, n.s. indicates not significant ($p$>0.05). (**c**) Overview of the Arpp21-CLIP experiment. (**d**) Enrichment of miRNA target genes in genes bound by Arpp21. Thymus transcriptome was defined using genes detected in wildtype RNA-seq dataset GSE242306 (GSM7757939, GSM7757940, GSM7757941). MiRNA target genes listed in miRDB version 6.0 (https://mirdb.org/download.html) and Arpp21 targets identified in this study were used. Target/non-target and Arpp21-bound/unbound genes were classified, and their association was tested using post-ANOVA Fisher-test. (**e**) Volcano plot of DEGs identified in mRNA-sequencing of DN2 thymocytes showing the −log10 (*P* value) plotted against the log2 (fold-change) (log2(FC)). Arpp21-target genes (red) identified by iCLIP are also shown. (**f**) Integrated genome view displaying Arpp21 iCLIP reads in the 3′-UTRs of *P2rx1* and *CD81*. (**g**) Representative contour plots of flow cytometry analyses of Roquin-1/2 expression in MEF cells transduced with retrovirus encoding inducible GFP-Arpp21, GFP-Arpp21short or GFP alone. Data are representative of two or more experiments. (**h**) Immunoblot analysis of Roquin1/2 in the indicated MEF cells overexpressed Arpp21. Gapdh serves as a loading control.

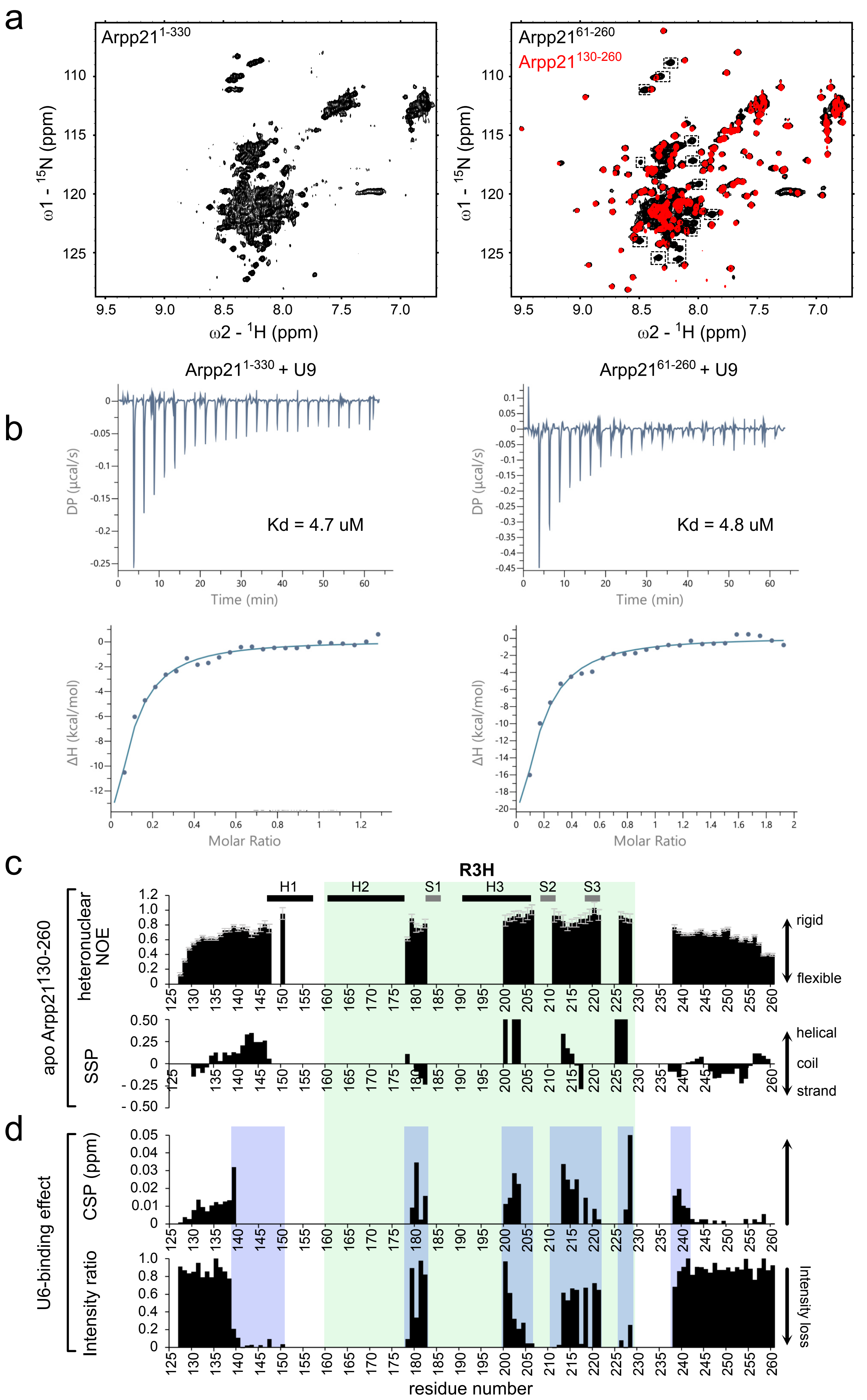


Supplementary Figure 4

**Supplementary Fig. 4. Structural determinants of Arpp21/RNA interaction.**
**(a)** HSQC spectrum of Arpp21$^{1\text{-}330}$ (left) and superimposed spectra (right) of Arpp21$^{61\text{-}260}$ (black) and Arpp21$^{130\text{-}260}$ (red). Representative and resolved signals belonging to the residues 61-129 are highlighted with boxes, in the right spectra. (**b**) ITC-monitored binding isotherms of Arpp21$^{1\text{-}330}$ (left) and Arpp21$^{130\text{-}260}$ (right) for U9 RNA. The estimated Kd's are shown.
(**c**) Backbone dynamics (above) and secondary structure propensities (SSP, below) for apo Arpp21$^{130\text{-}260}$, using $^{15}$N-heteronuclear NOE experiment and Ca & Cb chemical shift-derived secondary structures, respectively. PROSITE-predicted R3H domain is highlighted with a green shade. AlphaFold-predicted secondary structure components are depicted at the top. (**c**)The R3H domain showed highest heteronuclear NOE values ~0.9, suggesting a structured core, and less flexible N- and C-terminal sequences (residues 135-147 and 238-257) (**c, top panel**). The N-terminal region adjacent to the R3H is predicted to adopt helical propensity, while the C-terminal region is rather random coil, based on the backbone resonances of Ca and Cb (**c, bottom panel**). (**d**) Chemical shift perturbation (CSP, above) and NMR signal intensity changes (ratio, yellow) upon adding an equimolar U6 RNA to Arpp21$^{130\text{-}260}$, corresponding to the differences of red and blue spectra. Residues with either significant CSP or intensity loss are highlighted with blue shades.

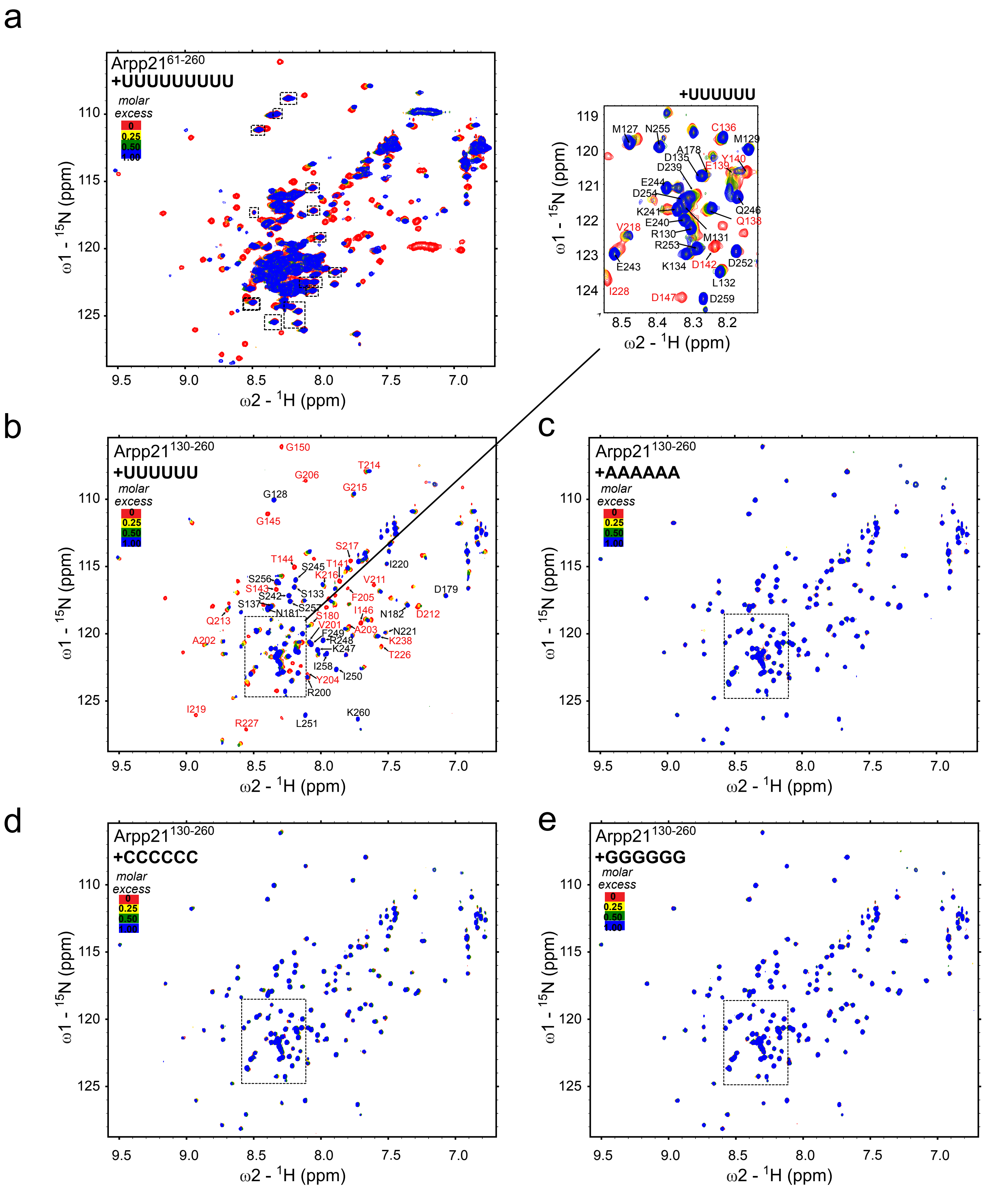


Supplementary Figure 5

**Supplementary Fig. 5. Analyzing intrinsic binding preferences of the Arpp21 R3h domain.**

(**a**) HSQC-monitored titration of Arpp21$^{61\text{-}260}$ with U9 RNA up to an equimolar concentration. (**b**) HSQC-monitored titration of Arpp21$^{130\text{-}260}$ with U6 RNA up to an equimolar concentration. Assigned resonances are annotated and the resonances with significant CSP or intensity loss, at the equimolar U9 RNA addition, are annotated in red. (**c-e**) HSQC-monitored titrations of Arpp21$^{130\text{-}260}$ with A6, C6, G6 RNAs, as indicated, respectively. The dotted boxes correspond to the region shown in **Fig. 4f**.

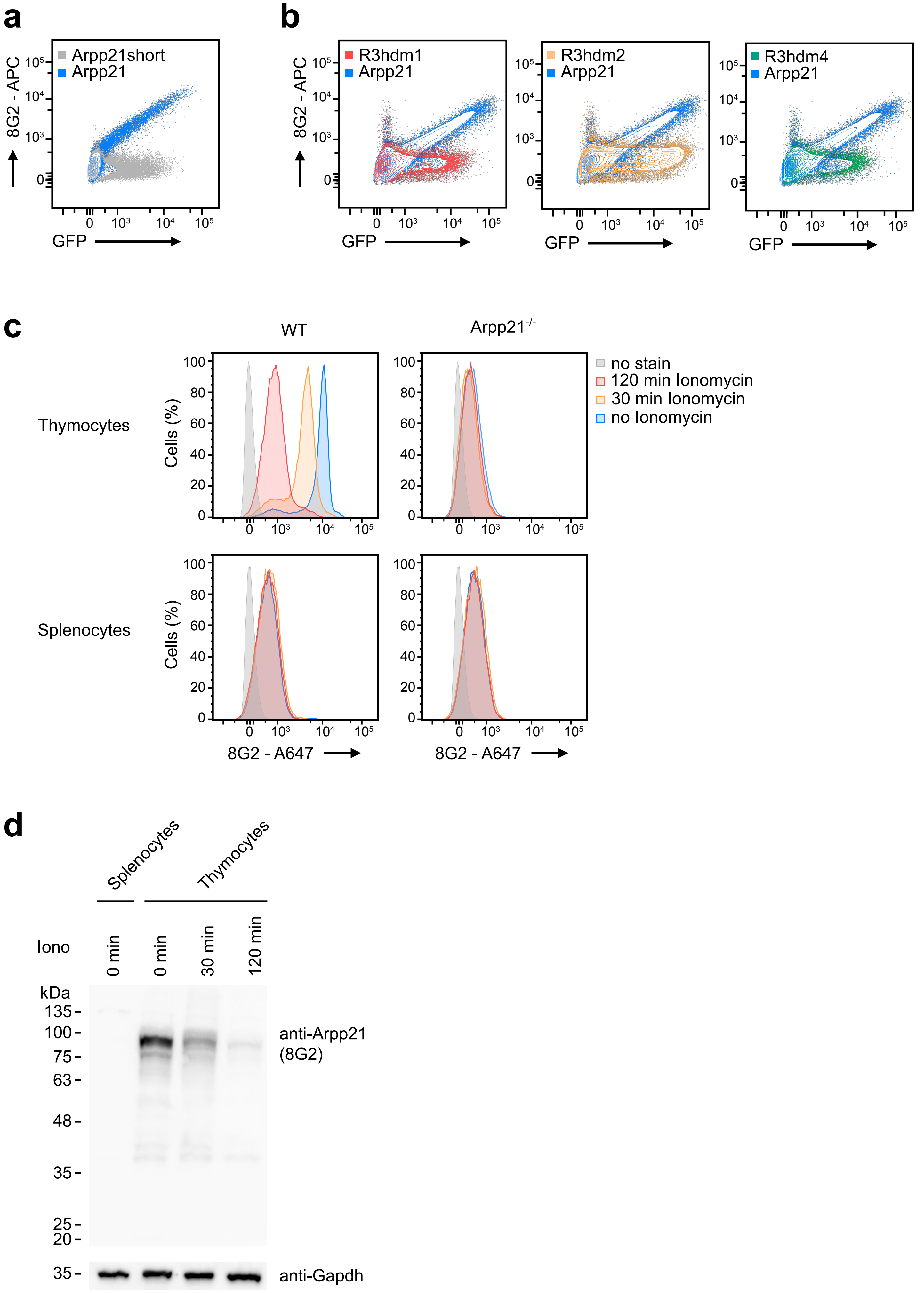


Supplementary Figure 6

**Supplementary Fig. 6. Evaluation of 8G2, a monoclonal antibody generated to specifically recognize the full-length Arpp21 protein.** (**a, b**) Overexpressed GFP-fusion proteins in 293T cells were used for subsequent flow cytometry analysis, the 8G2 supernatant detected Arpp21, but did not react to $Arpp21^{short}$ (**a**) or the paralogs R3hdm1, R3hdm2 or R3hdm4 (**b**). (**c**) Thymocytes and splenocytes from wildtype or Arpp21-deficient mice were treated with ionomycin for the indicated periods of time and subsequently analyzed by flow cytometry. (**d**) Aliquots from treated and untreated WT cells (**c**) were analyzed in Western blots.

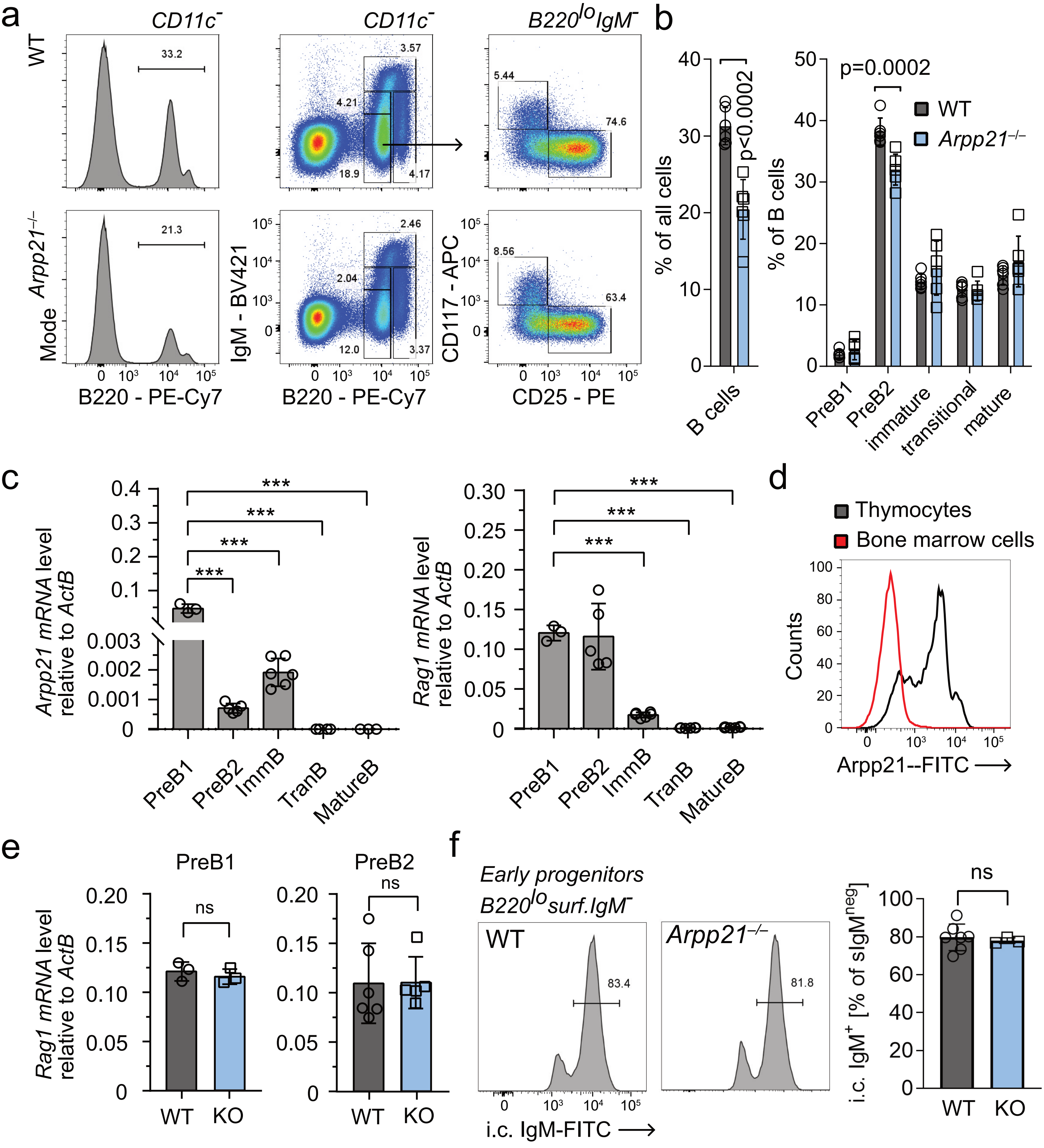


Supplementary Figure 7

**Supplementary Fig. 7. Characterization of the B cell compartment in Arpp21 KO mice.** (**a**) Representative histograms and dot plots showing the major B cell populations which were defined as follows: B cells–B220$^{+}$CD11c$^{-}$ and subsequently PreB1–B220$^{lo}$IgM$^{-}$CD117$^{+}$, PreB2– B220$^{lo}$IgM$^{-}$CD25$^{+}$, immature–B220$^{lo}$IgM$^{lo}$, transitional–B220$^{lo/hi}$IgM$^{hi,}$ mature–B220$^{hi}$IgM$^{lo/-}$ (**b**) Quantification of the data shown in (**a**). (**c**) Expression of Arpp21 (left) and Rag1 (right) transcripts in relation to ActB. (**d**) Representative histograms showing Arpp21 expression with 8G2 antibody in thymocytes and bone marrow cells. (**e**) Expression of Rag1 transcript in the indicated B cell progenitor populations. Data are representative of two independent experiments, each data point represents a single mouse. Statistical analysis was performed using two-way ANOVA followed by Sidak's multiple comparison test (**b**, **c**) or two-tailed t-test (**b** (left) and **e**); *** - p<0.001. (**f**) Intracellular IgM staining in the indicated B cell progenitor populations. n = 3 biological replicates.

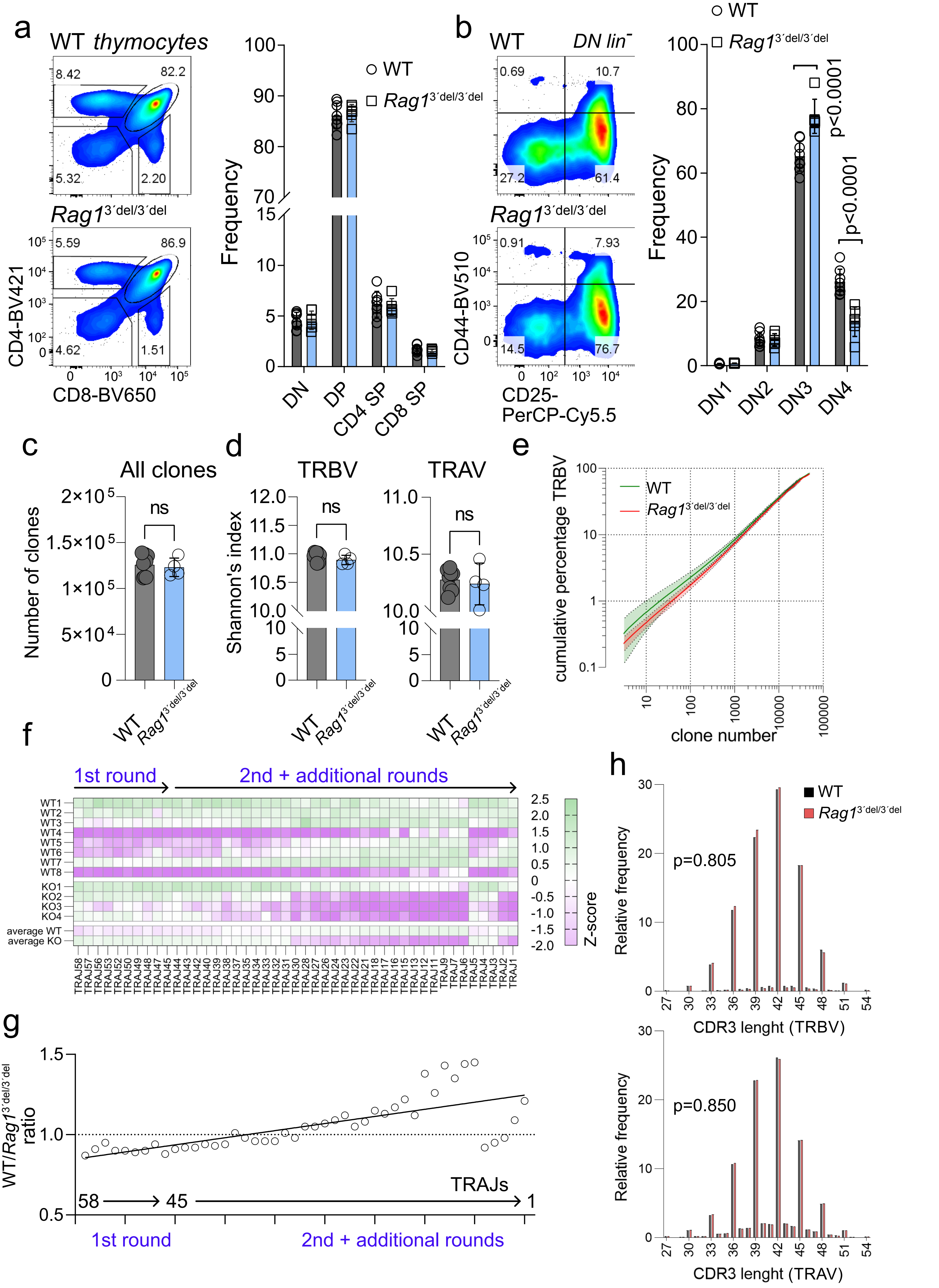


Supplementary Figure. 8

**Supplementary Fig. 8.** ***Rag1***$^{\mathbf{3'del/3'del}}$ **phenocopies Arpp21 deficiency in T cells.** (**a**) Representative plots (left) and statistical analysis (right) showing the frequency of major WT and KO thymocyte populations. (**b**) Representative plots (left) and statistical analysis (right) of major DN populations. (**c**) Number of individual TRBV and TRAV clones identified in peripheral CD4 T cells from WT and KO mice. (**d**) Shannon's diversity indices for TRBV and TRAV clones. (**e**) Cumulative percentage of the most frequent 50,000 clones. Solid lines indicate the mean of either combined WT and het mice (green) or KO mice (red), shaded areas indicate the standard deviation. (**f**) Heat map showing the z-score of the relative usage of all productive TRAJ segments in splenic CD4 T cells. Each line represents a single mouse, except for the last two where the average for WT and *Rag1*$^{3'del/3'del}$ is shown. (**g**) WT vs. KO ratio of usage of all productive TRAJ segments in relation to their chromosomal location. (h) Length distribution (measured in nucleotides) of all TRBV and TRAJ CDR3 elements detected in WT and KO T cells. Representative data from two independent experiments (**a**, **b**) or data from single experiments where results from all mice are shown (**c-h**). Each data point represents a single mouse (**a-d**). Statistical analysis was performed using two-way ANOVA followed by Sidak's multiple comparison test (**a**, **b**) or two-tailed t-test (**c-d**). Significance in (**a**, **b**) is indicated for $p<0.05$ only.

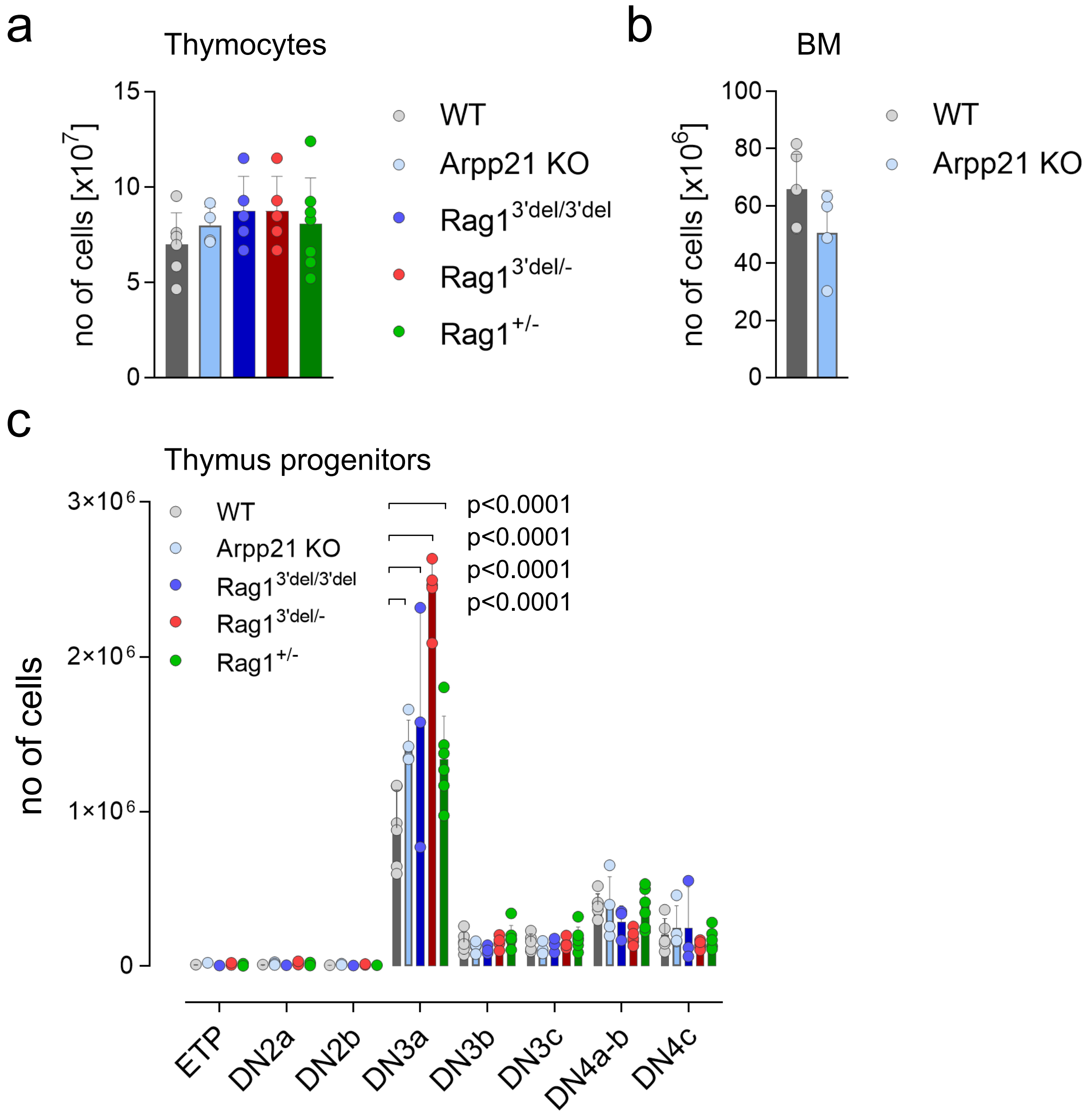


Supplementary Figure 9

**Supplementary Fig. 9. Cellularity of primary lymphoid organs in mice with indicated genotypes.** Total number of thymocytes (**a**) and bone marrow cells (**b**). (**c**) Number of ETP to DN4c thymic progenitor cells. Representative data of two independent experiments are shown. Each point represents a single mouse. Statistical analysis was performed using two-way ANOVA followed by Sidak's multiple comparison test (**a**, **c**) or two-tailed t-test (**b**).

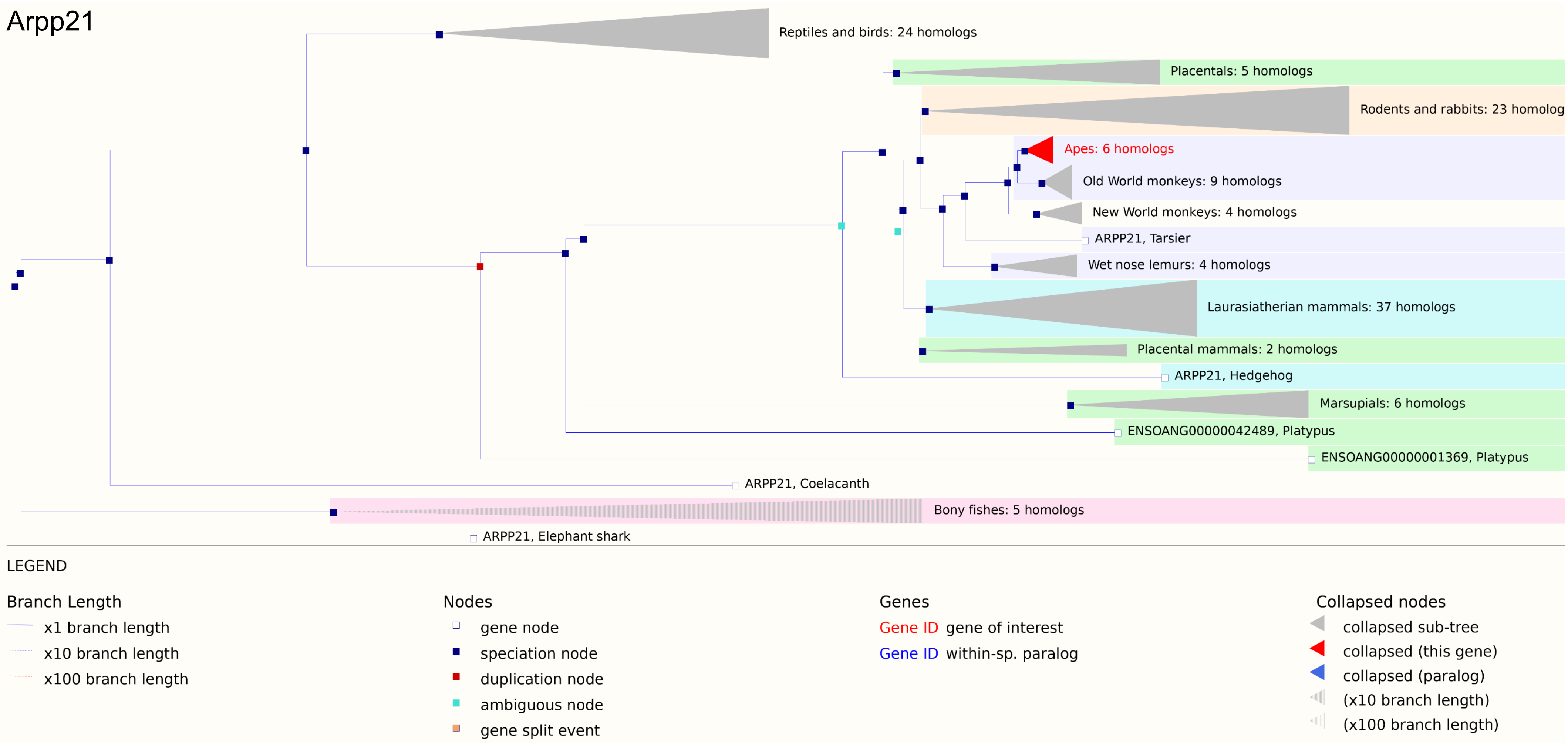


Supplementary Figure 10

**Supplementary Fig. 10. Phylogenetic tree of Arpp21.** In evolution, Arpp21 first appeared in Chondrichthyes (represented here by the elephant shark), which correlates with the emergence of the adaptive immune system. Graph adapted from Ensembl release 109.

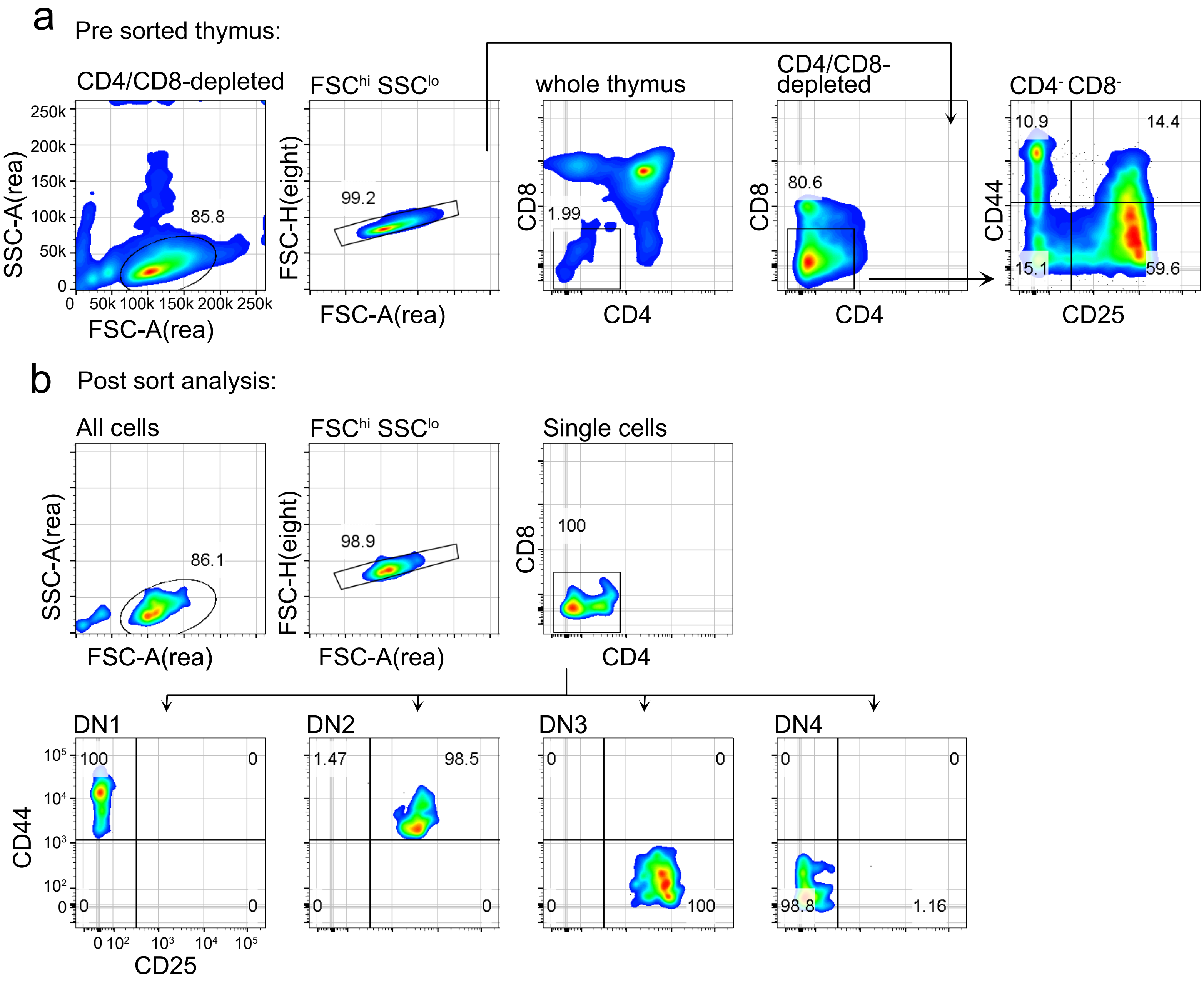


Supplementary Figure. 11

**Supplementary Fig. 11. Gating and sorting strategy of T cells and their progenitors.** (a) FSC and SSC parameters were used to select cells of the correct size and to exclude cell doublets. Double negative progenitors were defined based on their CD44 and CD25 expression and sorted from CD4 and CD8 depleted thymus. (b) Schematic representation of post-sorted thymic progenitors.

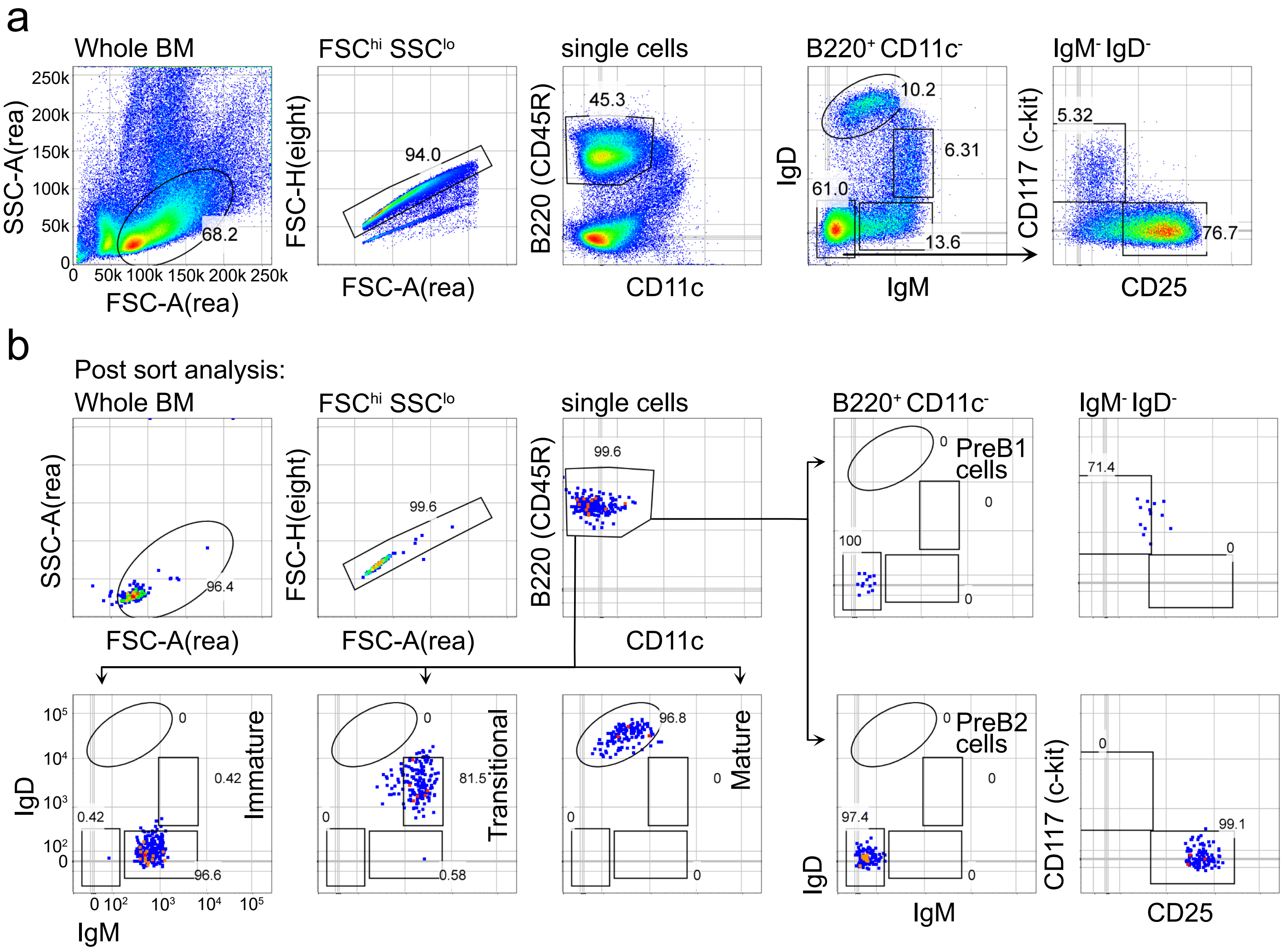


Supplementary Figure. 12

**Supplementary Fig. 12. Gating and sorting strategy for the identification and purification of bone marrow B-cell precursors.** (a) FSC and SSC parameters were used to identify cells of the correct size and to exclude cell doublets. B cell precursors were defined as $B220^+$ and $CD11c^-$. Early progenitors are negative for IgM and IgD and are either CD117 (PreB1) or CD25 (PreB2) positive. Other populations were defined as $IgM^+$ $IgD^-$ (immature), $IgM^+$ $IgD^+$ (transitional) and $IgM^{lo}$ $IgD^{hi}$ (mature) (b) Schematic of post-sorting analysis of bone marrow B cell precursors.

**a**

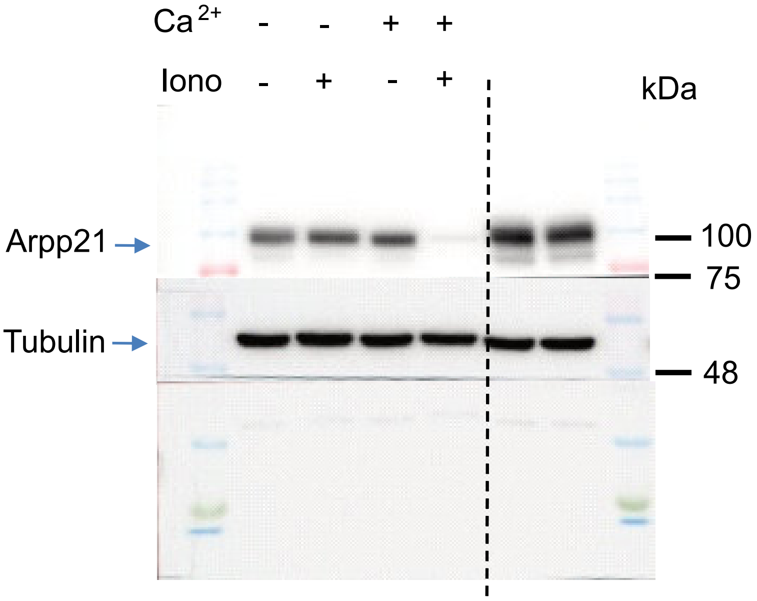


**b**

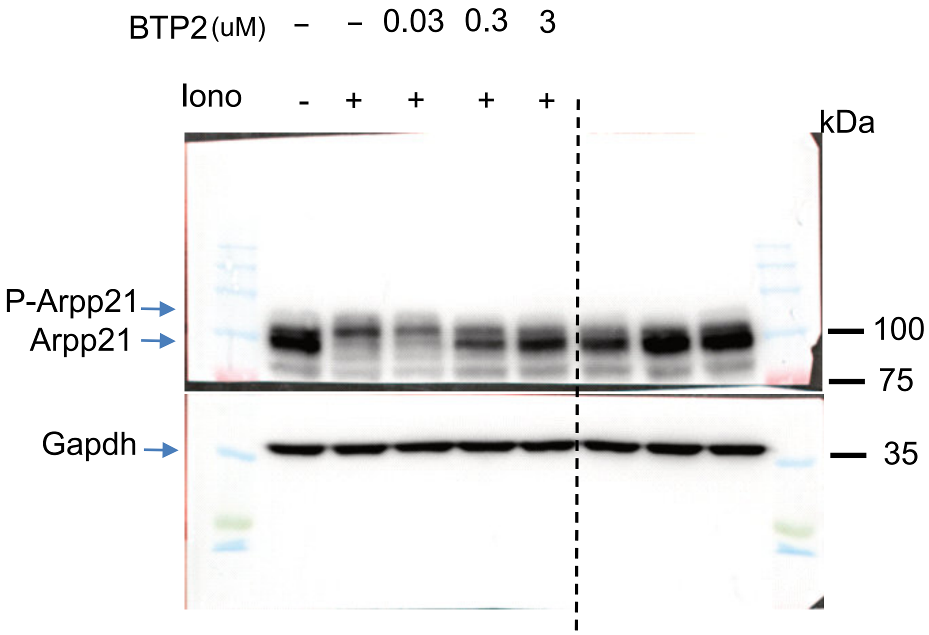


**e**

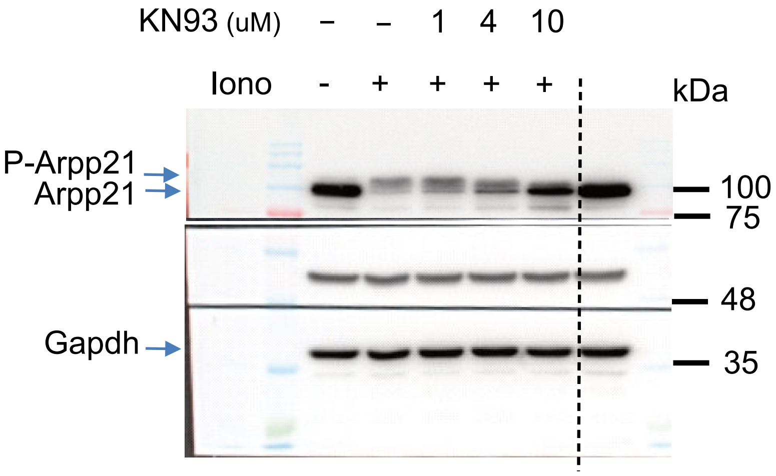


uncropped Supplementary Figure 2

h

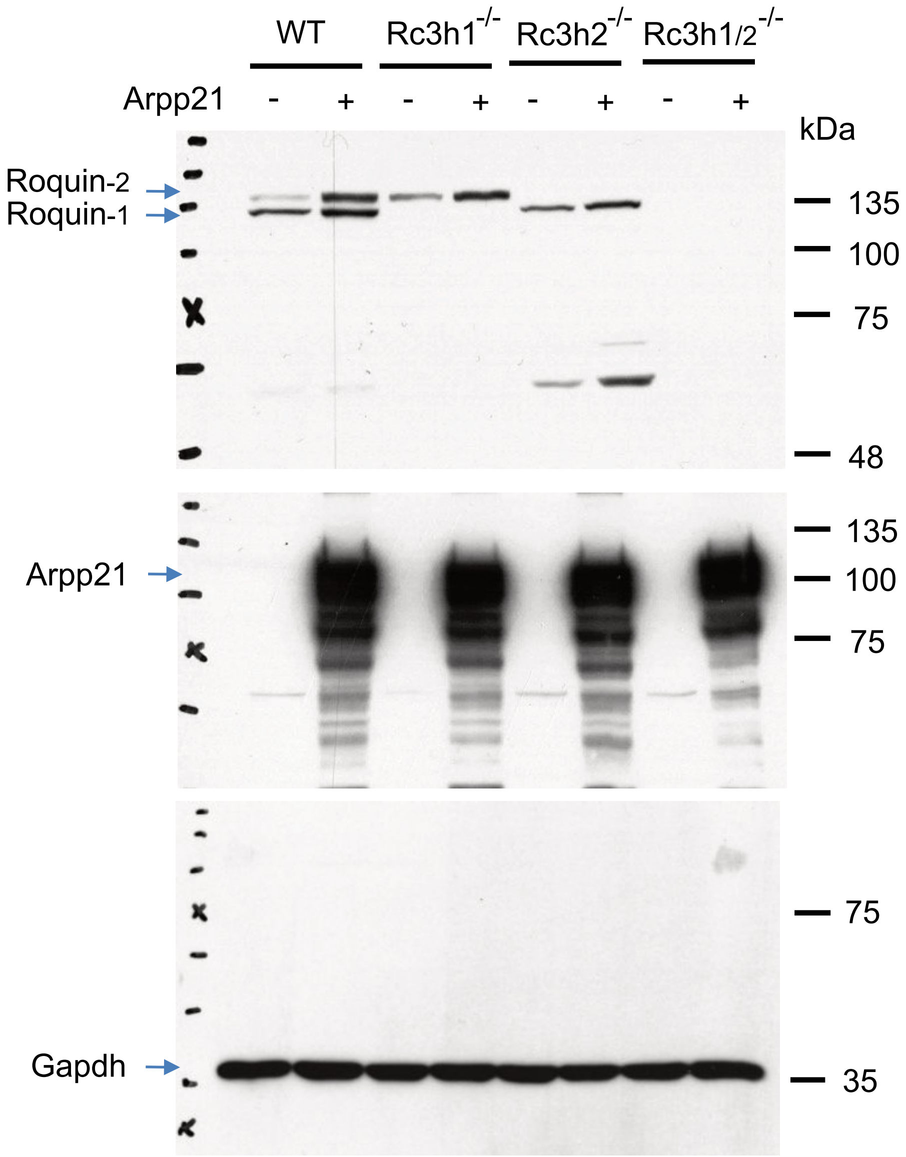


uncropped Supplementary Figure 3

**Uncropped Blots of Supplemental Material.** The following blots show uncropped images of Supplementary Fig.2a, b, e, and of Supplementary Fig. 3h, as well as of Supplementary data Fig. 6d.
