# Supplementary material for: The thymocyte-specific RNA-binding protein Arpp21 provides TCR repertoire diversity by binding to the 3’-UTR and promoting Rag1 mRNA expression
Source: Nat Commun. 2024 Mar 11;15:2194. doi: 10.1038/s41467-024-46371-z (PMC10928157; doi:10.1038/s41467-024-46371-z)
Supplement: Supplementary file 3 — Description of Additional Supplementary Files [file 41467_2024_46371_MOESM3_ESM.pdf]

Description of Additional Supplementary Files

Supplementary Data 1: Mass spectrometry data used to identify RNA-binding proteins in thymocyte OOPS experiments.

**Sheet name**	**Description**
Identified crosslink site	OpenNuXL crosslink site identification results at 1% spectral FDR for thymocytes or T cells and manually validated crosslink spectra for IP experiements. Additional columns specifying if the peptide falls in a known domain (UniProt) and GO molecular function of the leading protein.
Thymocyte RBPs	Results of protein enrichment Student's *t*-test between OOPS purified crosslinked and non-crosslinked cells. Additioanal columns were added to asign if a protein from the protein groups was identified in EuRBPDB database or crosslink site localization experiments in thymocytes or T cells.
Thymocyte-identified RBPs	Comparison of RBPs identified in OOPS experiments from thymocytes and CD4 T cells (Hoefig *et al.*, 2021).
