# Supplementary material for: The thymocyte-specific RNA-binding protein Arpp21 provides TCR repertoire diversity by binding to the 3’-UTR and promoting Rag1 mRNA expression
Source: Nat Commun. 2024 Mar 11;15:2194. doi: 10.1038/s41467-024-46371-z (PMC10928157; doi:10.1038/s41467-024-46371-z)
Supplement: Supplementary file 5 — Reporting Summary [file 41467_2024_46371_MOESM5_ESM.pdf]

nature portfolio

Corresponding author(s): Vigo Heissmeyer
Last updated by author(s): 2024/01/28

Reporting Summary

Nature Portfolio wishes to improve the reproducibility of the work that we publish. This form provides structure for consistency and transparency in reporting. For further information on Nature Portfolio policies, see our Editorial Policies and the Editorial Policy Checklist.

Statistics

For all statistical analyses, confirm that the following items are present in the figure legend, table legend, main text, or Methods section.

n/a	Confirmed	
☐	☒	The exact sample size (*n*) for each experimental group/condition, given as a discrete number and unit of measurement
☐	☒	A statement on whether measurements were taken from distinct samples or whether the same sample was measured repeatedly
☐	☒	The statistical test(s) used AND whether they are one- or two-sided<br>*Only common tests should be described solely by name; describe more complex techniques in the Methods section.*
☒	☐	A description of all covariates tested
☐	☒	A description of any assumptions or corrections, such as tests of normality and adjustment for multiple comparisons
☐	☒	A full description of the statistical parameters including central tendency (e.g. means) or other basic estimates (e.g. regression coefficient) AND variation (e.g. standard deviation) or associated estimates of uncertainty (e.g. confidence intervals)
☐	☒	For null hypothesis testing, the test statistic (e.g. *F*, *t*, *r*) with confidence intervals, effect sizes, degrees of freedom and *P* value noted<br>*Give P values as exact values whenever suitable.*
☒	☐	For Bayesian analysis, information on the choice of priors and Markov chain Monte Carlo settings
☒	☐	For hierarchical and complex designs, identification of the appropriate level for tests and full reporting of outcomes
☒	☐	Estimates of effect sizes (e.g. Cohen's *d*, Pearson's *r*), indicating how they were calculated

Our web collection on statistics for biologists contains articles on many of the points above.

Software and code

Policy information about availability of computer code

Data collection	Mass spectrometric acquisition was done with Thermo Scientific Xcalibur 4.4.16.14<br>Flow cytometry data were collected on FACS DIVA version 8 or SpectroFlo.<br>qPCR data were collected with LightCycler 480 Software.<br>Total RNA was processed using the SMARTer TCR a/b Profiling Kit (Takara). RNA was quality checked using Qubit and TapeStation and sequenced using the 600 cycle kit on the Illumina MiSeq platform.<br>PCR assay for V(D)J recombination data were collected on Quantum ST4 imaging system (Vilber Lourmat).<br>Reporter assay data were collected on GloMax Dual-Luciferase Reporter Assay System (Promega).<br>NMR data were acquired on 600MHz Bruker AV III (cryo-TCI) and 1.2GHz Bruker Avance III HD (3mm cryo-TCI).<br>ITC experiments were performed using MicroCal PEAQ-ITC (Malvern Panalytical).
Data analysis	OOPS protein enrichment analysis was performed with MaxQuant 2.1.4.0 and Perseus 2.0.6.0<br>Crosslink site identification was done with OpenNuXL node of OpenMS 3.0.0<br>iClip data were analyzed with STAR v2.7.6a.<br>RNA-seq data were analyzed with kallisto v0.46.<br>TCR Seq data were analysed using the MIXCR pipeline to assemble the final CDR3. Advanced analyses to compare VJ gene usage between samples and to determine Shannon's diversity and frequency changes in the repertoire between samples were performed as previously described (ref 47, 48).<br>Flow Cytometry Data with: FlowJo V10.6.0 (BD Bioscience).<br>SDS-PAGE images were analyzed with ImageJ (NIH).<br>data and statistical analysis was performed with Prism 9.4.1 (Graphpad).

NMR spectra were processed by TOPSPIN3.5 (Bruker) and NMRpipe 11.0, and analyzed using NMRFAM-SPARKY 1.2 (powered by Sparky 3.115).
ITC titrations were analyzed using MicroCal PEAQ-ITC Analysis Software v1.41 (Malvern Panalytical).

For manuscripts utilizing custom algorithms or software that are central to the research but not yet described in published literature, software must be made available to editors and reviewers. We strongly encourage code deposition in a community repository (e.g. GitHub). See the Nature Portfolio guidelines for submitting code & software for further information.

Data

Policy information about availability of data

All manuscripts must include a data availability statement. This statement should provide the following information, where applicable:

- Accession codes, unique identifiers, or web links for publicly available datasets
- A description of any restrictions on data availability
- For clinical datasets or third party data, please ensure that the statement adheres to our policy

RNA-seq and iClip datasets have been deposited at Gene Expression Omnibus (GEO) site under the accession number GSE198798; TCR repertoire sequencing data is available at GEO site under the number GSE226368 and GSE226369. The proteomic data have been submitted to MassIVE under the accession number MSV000091606 (http://massive.ucsd.edu/ProteoSAFe/status.jsp?task=487f90eab5e54492b0693628332bb555 ; Username: MSV000091606_reviewer
Pass: K8kVSHh9Lp3bhHX)

All data provided in this article are available from the corresponding author upon reasonable request.

Research involving human participants, their data, or biological material

Policy information about studies with human participants or human data. See also policy information about sex, gender (identity/presentation), and sexual orientation and race, ethnicity and racism.

Reporting on sex and gender	NA
Reporting on race, ethnicity, or other socially relevant groupings	NA
Population characteristics	NA
Recruitment	NA
Ethics oversight	*Identify the organization(s) that approved the study protocol.* NA

Note that full information on the approval of the study protocol must also be provided in the manuscript.

Field-specific reporting

Please select the one below that is the best fit for your research. If you are not sure, read the appropriate sections before making your selection.

[x] Life sciences [ ] Behavioural & social sciences [ ] Ecological, evolutionary & environmental sciences

For a reference copy of the document with all sections, see nature.com/documents/nr-reporting-summary-flat.pdf

Life sciences study design

All studies must disclose on these points even when the disclosure is negative.

Sample size	No sample size calculation was performed. Sample size was determined according to common practice in the field and our own experience (Vogel et al., 2013; PMID: 23583643) and was equal to sample sizes used in previous publications. Whenever possible at least 3 biological replicates were analysed. In vivo experiments were performed twice whenever possible at least 6 biological replicates were used to ensure reproducibility.

Data exclusions | no data exclusion was performed

Replication | Data from in vitro experiments and immune phenotyping are representative of at least two independent experiments with similar results. For in vivo experiments that were only performed once, at least six biological replicates were used and no animals have been excluded.

Randomization | Both male and female mice (6 weeks-1 year of age as indicated in the figure legends) were analyzed with sex- and aged-matched control mice, whereas, whenever possible, littermates were used as controls. All mice were grouped according to genotype. Randomization was not important since the the flow cytometric analyses are unbiased.

Blinding | Mice were grouped according to genotype and comparisons were made between different genotypes. Blinding was not possible, since different genotypes displayed distinct phenotypes and cellularities, which need to be accounted for during sample preparation. Blinding was not performed, because the analyses did not include subjective assessments.

Reporting for specific materials, systems and methods

We require information from authors about some types of materials, experimental systems and methods used in many studies. Here, indicate whether each material, system or method listed is relevant to your study. If you are not sure if a list item applies to your research, read the appropriate section before selecting a response.

Materials & experimental systems

n/a	Involved in the study	
☐	☒	Antibodies
☐	☒	Eukaryotic cell lines
☒	☐	Palaeontology and archaeology
☐	☒	Animals and other organisms
☒	☐	Clinical data
☒	☐	Dual use research of concern
☒	☐	Plants

Methods

n/a	Involved in the study	
☒	☐	ChIP-seq
☐	☒	Flow cytometry
☒	☐	MRI-based neuroimaging

Antibodies

Antibodies used

Following commercial FACS antibodies were used:
anti-CD3-APC-Fire810(cl. 17A2, Cat. 100268, Biolegend)
anti-CD4-BV421(cl. RM4-5, Cat. 100543, Biolegend)
anti-CD4-BV786 (cl. RM4-5, Cat. 563727, BD Pharmingen)
anti-CD4-BV421(cl. RM4-5, Cat. 100543, Biolegend)
anti-CD4-BUV661 (cl. GK1.5, Cat. 612974, BD Pharmingen)
anti-CD8a-FITC (cl. 53-6.7, Cat. 48-0081-82, eBioscience)
anti-CD8a-BV650 (cl. 53-6.7, Cat. 100741, Biolegend)
anti-CD8a-BV750 (cl. 53-6.7, Cat. 747134, BD Pharmingen)
anti-CD11b-BV480 (cl. M1/70, Cat. 566117, BD Pharmingen)
anti-CD11c-BUV737 (cl. N418, Cat. 367-0114-82, eBioscience)
anti-CD11c-FITC (cl. N418, Cat. 11-0114-82, eBioscience)
anti-CD19-BV711 (cl. 6D5, Cat. 115555, Biolegend)
anti-CD24-BV605 (cl. M1/69, Cat. 101827, Biolegend)
anti-CD24-PerCP-eFluor 710 (cl. M1/69, Cat. 46-0242-82, eBioscience)
anti-CD25-PE (cl. PC61, Cat. 553866, BD Pharmingen)
anti-CD25-PE-Cy7 (cl. 3C7, Cat. 101916, BD Pharmingen)
anti-CD25-PerCP-Cy5.5 (cl. PC61.5, Cat. 45-0251-82, invitrogen)
anti-CD25-Alexa Fluor488 (cl. PC61.5, Cat. 53-0251-82, invitrogen)
anti-CD28-PE (cl. 37.51, Cat. 102106, Biolegend)
anti-CD28-BB700 (cl. 37.51, Cat. 566512, BD Pharmingen)
anti-CD43-PE-Cy5 (cl. 1B11, Cat. 121216 Biolegend)
anti-CD44-BV421 (cl. IM7, Cat. 103039, Biolegend)
anti-CD44-BV510 (cl. IM7, Cat. 103043, Biolegend)
anti-CD44-BV570 (cl. IM7, Cat. 103037, Biolegend)
anti-CD62L-eFluor 450 (cl. MEL14, Cat. 48-0621-82, eBioscience)
anti-CD69-PE-Cy7 (cl. H1.2F3, Cat. 104512, Biolegend)
anti-CD45R (B220)-PE-Cy7 (cl. RA3-6B2, Cat. 25-0452-82, eBioscience)
anti-CD45R (B220)-BV605 (cl. RA3-6B2, Cat. 103243, Biolegend)
anti-CD117-APC (cl. 2B8, Cat. 553356, BD Pharmingen)
anti-CD117-BV421 (cl. QA17A09, Cat. 155109, BD Pharmingen)
anti-CD127-BUV737 (cl. A7R34, Cat. 367-1271-82, eBioscience)
anti-CD135-PE-Cy7 (cl. 3C7, Cat. 101916, Biolegend)
anti-CD161-BUV563 (cl. PK136, Cat. 741233, BD Pharmingen)
anti-IgD-PE-Dazzle 594 (cl. 11-26c.2a, Cat. 405742, Biolegend)

anti-IgM-BV421 (cl. RMM-1, Cat. 406517, Biolegend)
anti-IgM-BUV615 (cl. II/41, Cat. 751479, BD Pharmingen)
anti-TCRbeta-PE-Cy7 (cl. H57-697, Cat. 109221, Biolegend)
anti-TCRbeta-BUV395 (cl. H57-697, Cat. 742484, BD Pharmingen)
anti-Ter119-BUV496 (cl. TER-119, Cat. 741079, BD Pharmingen)
anti-NK1.1-BUV563 (cl. PK136, Cat. 741233, BD Pharmingen)
anti-Helios-PE (cl. 22F6, Cat. 137216, Biolegend)
anti-TCRgd-APC (cl. GL3, Cat. 118116, Biolegend)
anti-TCRgd-Super Bright 780 (cl. GL3, Cat. 78-5711-82, eBioscience)
anti-Sca1-BV650 (cl. D7, Cat. 108143, Biolegend)
anti-Gr1-SuperBright645 (cl. RB6-8C5, Cat. 64-5931-82, eBioscience)
anti-Gr1-PerCP-Cy5.5 (cl. RB6-8C5, Cat. 45-5931-80, eBioscience)
anti-MHC Class II (I-Ab)-eFluor 450 (cl. M5/114.15.2, Cat. 48-5321-82, eBioscience)
anti-PAX5-APC (cl. 1H9, Cat. 17-9918-80, eBioscience)

goat-anti-Rat-AF647 (cl. poly4054, Cat. 405416, Biolegend)

Antibodies used in Western bolt and IP:
anti-Arpp21 (polyclonal 1:500, Cat. 11829-1-AP, Proteintech)
anti-Arpp21 (monoclonal, 1:1000)
anti-Roquin-1/2 (1:10, cl. 3F12, inhouse production)
anti-Rag1 (1:2000, Cat. ab172637, abcam)
anti-Ubiquitin (1:1000, Cat. BML-PW8810-0100, Enzo Life Sciences)
anti-Gapdh (1:10000, Cat. CB1001, Merck Millipore)
anti-Tubulin (1:1000, Cat. sc-23948, Santa Cruz Biotechnology)
HRP-anti-Rat (1:3000, Cat. 7077, Cell Signaling)
HRP-anti-Mouse (1:3000, Cat. 7076, Cell Signaling)
HRP-anti-Rabbit (1:3000, Cat. 7074, Cell Signaling)

Validation

All above mentioned antibodies are well validated commercial clones routinely QC'ed by the manufacturer. Please refer to the data sheets on the vendors' website for technical information. We have provided a full Supplementary Fig. 6 for the Validation of the Arpp21 8G2 monoclonal antibody.

Eukaryotic cell lines

Policy information about cell lines and Sex and Gender in Research

Cell line source(s)

HEK 293T, Hela cells were obtained from the American Type Culture Collection (ATCC).
Mouse Embryonic Fibroblast (MEF) cells were generated, immortalized, genetically manipulated and cloned in-house.

Authentication

None of the cell lines were authentificated.

Mycoplasma contamination

All used cell lines are tested negative for mycoplasma.

Commonly misidentified lines
(See ICLAC register)

No cell lines that have been reported as misidentified have been used in this study.

Animals and other research organisms

Policy information about studies involving animals; ARRIVE guidelines recommended for reporting animal research, and Sex and Gender in Research

Laboratory animals

All animals used were on a C57BL/6 background and in the age of 6 weeks to 1 year old.
Mice were housed in a specific-pathogen-free barrier facility under a 12h/12h dark/light regime at 20-24℃, at a humidity of 45-65%, and air exchange rate of 10-15 times per hour.
For immune phenotyping, male and female 6 weeks to 1 year old mice were used.

Following mouse strains were used (sex and age are indicated in the manuscript):
- Arpp21 KO
- Rag1 KO
- Rag1 3’-UTR KO
- OTI
- CamK4 KO
- Stim1fl/fl, Stim2fl/fl, vavcre
- Stim1fl/fl, Stim2fl/fl
- WT

Wild animals

This study did not involve wild animals.

Reporting on sex	All animal experiments were involved with male and female mice, and sex was not considered in this study design.
Field-collected samples	This study did not involve samples collected from the field.
Ethics oversight	All experimental procedures were performed following the rules and regulations approved by the local government (Regierung von Oberbayern reference number 55.2-2532.Vet_02-19-68).

Note that full information on the approval of the study protocol must also be provided in the manuscript.

Flow Cytometry

Plots

Confirm that:

- [x] The axis labels state the marker and fluorochrome used (e.g. CD4-FITC).
- [x] The axis scales are clearly visible. Include numbers along axes only for bottom left plot of group (a 'group' is an analysis of identical markers).
- [x] All plots are contour plots with outliers or pseudocolor plots.
- [x] A numerical value for number of cells or percentage (with statistics) is provided.

Methodology

Sample preparation	Thymocytes, bone marrow cells and splenocytes were mechanically disrupted and erythrocytes were removed. Single cells suspensions were stained with live/dead dyes and the indicated antibodies.
Instrument	LSR Fortessa (BD Bioscience) was used for data collection.<br>FACSAria Fusion (BD Bioscience) was used for cell sorting.
Software	Flow Cytometry data were analyzed using flowJo v10.6.0
Cell population abundance	1-2 million cells were measured in flow cytometry analysis.
Gating strategy	Cells were gated for lymphocyte population (SSC-A/FSC-A), single cells (SSC-W/SSC-H and FSC-W/FSC-H) and living cells using Fixable Blue Viability Dye, Fixable Viability Dye eF780 or DAPI.

- [x] Tick this box to confirm that a figure exemplifying the gating strategy is provided in the Supplementary Information.
